# Effect of Rice Straw and Stubble Burning on Soil Physicochemical Properties and Bacterial Communities in Central Thailand

**DOI:** 10.3390/biology12040501

**Published:** 2023-03-26

**Authors:** Noppol Arunrat, Sukanya Sereenonchai, Chakriya Sansupa, Praeploy Kongsurakan, Ryusuke Hatano

**Affiliations:** 1 Faculty of Environment and Resource Studies, Mahidol University, Nakhon Pathom 73170, Thailand; 2 Department of Biology, Faculty of Science, Chiang Mai University, Chiang Mai 50200, Thailand; 3 Graduate School of Fisheries and Environmental Sciences, Nagasaki University, Nagasaki 852-8521, Japan; 4 Laboratory of Soil Science, Research Faculty of Agriculture, Hokkaido University, Sapporo 060-8589, Japan

**Keywords:** paddy field, soil organic carbon, soil total nitrogen, microbial diversity, fire

## Abstract

**Simple Summary:**

Fire is traditionally used by farmers for clearing fields when the fallow period is short. Soil chemical properties changed significantly after burning and returned to prefire levels after 1 year. *Bacillus*, HSB OF53-F07, *Conexibacter*, and *Acidothermus* abundances increased immediately after burning and then significantly declined, with lower levels 1 year after burning. *Anaeromyxobacter* and Candidatus *Udaeobacter* dominated at 1 year after burning. Burning under high soil moisture conditions and within a very short time caused no effect to the bacterial soil communities.

**Abstract:**

Rice straw and stubble burning is widely practiced to clear fields for new crops. However, questions remain about the effects of fire on soil bacterial communities and soil properties in paddy fields. Here, five adjacent farmed fields were investigated in central Thailand to assess changes in soil bacterial communities and soil properties after burning. Samples of soil prior to burning, immediately after burning, and 1 year after burning were obtained from depths of 0 to 5 cm. The results showed that the pH, electrical conductivity, NH_4_-N, total nitrogen, and soil nutrients (available P, K, Ca, and Mg) significantly increased immediately after burning due to an increased ash content in the soil, whereas NO_3_-N decreased significantly. However, these values returned to the initial values. Chloroflexi were the dominant bacteria, followed by Actinobacteria and Proteobacteria. At 1 year after burning, Chloroflexi abundance decreased remarkably, whereas Actinobacteria, Proteobacteria, Verrucomicrobia, and Gemmatimonadetes abundances significantly increased. *Bacillus*, HSB OF53-F07, *Conexibacter*, and *Acidothermus* abundances increased immediately after burning, but were lower 1 year after burning. These bacteria may be highly resistant to heat, but grow slowly. *Anaeromyxobacter* and Candidatus *Udaeobacter* dominated 1 year after burning, most likely because of their rapid growth and the fact that they occupy areas with increased soil nutrient levels after fires. Amidase, cellulase, and chitinase levels increased with increased organic matter levels, whereas β-glucosidase, chitinase, and urease levels positively correlated with the soil total nitrogen level. Although clay and soil moisture strongly correlated with the soil bacterial community’s composition, negative correlations were found for β-glucosidase, chitinase, and urease. In this study, rice straw and standing stubble were burnt under high soil moisture and within a very short time, suggesting that the fire was not severe enough to raise the soil temperature and change the soil microbial community immediately after burning. However, changes in soil properties due to ash significantly increased the diversity indices, which was noticeable 1 year after burning.

## 1. Introduction

Rice straw comprises a major agricultural residue in Thailand. However, large amounts of rice straw are left in fields after harvesting, which are then often burned to prepare the land for the following crops [1,2]. The production of rice straw in Thailand has been estimated at over 20 million tons per year [3]. Among the rice cultivation areas in Thailand, the central region has the highest potential, with two harvests a year. According to the agricultural statistics of Thailand, the total area of the first rice harvest in central Thailand was 1.31 million ha in the crop year of 2020/2021 (3.74 tons ha^−1^ in average yield), whereas the second rice harvest area covered 0.54 million ha (4.33 tons ha^−1^ in average yield) [4]. This could be explained by its location in the floodplains of the Chao Phraya river basin, which facilitates water availability, thus, encouraging farmers to prepare their lands for the following crops as soon as possible. In this sense, burning is the method of choice for rapidly eliminating rice straw and stubble. If left in the field, rice straw and stubble can represent obstacles for land preparation, and using fire is also the most efficient method for controlling weeds and pests [5,6].

Direct burning in fields significantly changes the soil temperature, moisture, and organic matter content, especially in the topsoil layer [7,8], resulting in an abrupt decline in microbial biomass and diversity [9]. Mickovski [10] reported that burning rice straw and stubble resulted in an increase in soil temperatures by up to 50–70 °C in the uppermost 0 to 3 cm of soil, causing a 77% decrease in heterotrophic microorganisms. Biederbeck et al. [11] also found that bacterial populations in the topsoil layer (0–2.5 cm) were reduced by >50% in rice straw burning areas compared to areas where the rice straw remained in the fields. Furthermore, Kumar et al. [12] reported that paddy straw burning reduced the populations of bacteria, fungi, actinomycetes, phosphate-solubilizing microorganisms, potassium-solubilizing microorganisms, cellulose, and microbial enzymes.

Changes to the physical and chemical properties of soil after burning also affect the structure of soil bacterial communities due to the properties of rice straw ash [13]. Duan et al. [14] stated that rice straw ash is alkaline and mainly contains potassium oxide and silicon dioxide; silicon plays a crucial role as a biological stimulant for plant growth [15]. The ash causes changes in the soil pH, affecting the abundance of soil microorganisms. Guo et al. [16] and Zhao et al. [17] reported that changing the soil pH resulted in changes in the abundance of Acidobacteria in rice–wheat cropping systems, whereas the abundances of Proteobacteria, Gemmatimonadetes, and Nitrospirae in soil were modified due to changes in nitrogen, phosphorus, and potassium levels. Avoiding the burning of rice straw by incorporating it into the soil can improve soil nutrient contents by increasing carbon and nitrogen concentrations, thus, affecting the structure of soil bacterial communities. In a study by Zhao et al. [17], the population of Proteobacteria increased following the addition of rice straw due to an increase in soil nitrogen levels. Currently, the incorporation of rice straw into soil is not always cost-effective for farmers in central Thailand, and, thus, burning is the most effortless and cheapest practice for clearing fields when the fallow period is short. However, the effects of burning rice straw and stubble on soil properties and soil bacterial communities remain poorly understood. We hypothesized that soil nutrients would increase, but that soil bacterial communities would decrease after a fire. The objectives of the present study were as follows: (1) to determine the effects of fire on soil organic carbon (SOC), soil total nitrogen (STN), and soil nutrients in paddy fields; (2) to examine changes in the composition and diversity of soil bacterial communities as a result of rice straw and stubble burning; and (3) to analyze the relationship between soil nutrients and soil bacterial communities. The results of this study provide the scientific knowledge for the minimization of postfire risks in paddy fields, which could contribute to implementing suitable management practices for maintaining biodiversity and ecosystem functioning.

## 2. Materials and Methods

### 2.1. Study Site

The study was conducted in the Taluk subdistrict, Sapphaya district, Chainat province, central Thailand. The area is located in the floodplain of the Chao Phraya river basin and has a tropical savanna (Köppen ‘Aw’) climate, with an average annual temperature of 27.8 °C. April and May are the hottest months, with maximum temperatures in the range of 38–40 °C, whereas the lowest temperatures occur during December–February (18–23 °C). The average annual precipitation ranges from 1000 to 1200 mm. The highest precipitation is usually recorded in September, with a total monthly precipitation of 200–300 mm and 10–18 rainy days. The soil belongs to the Chainat series (Cn), which consists of fine, mixed, active, nonacidic, and isohyperthermic Aeric (Vertic) Endoaquepts, and is predominantly dark grayish brown and dark gray. The pH ranges from 5.5 to 6.5, and the soil texture is characterized by silty clay or clay loam. The slopes range from 0 to 2%.

### 2.2. Field Management Practices and Fire Measurements

To avoid the effects of variation in environmental conditions, five adjacent farmed fields were investigated in the Taluk subdistrict. The “RD 43” (95 days), “RD 57” (110 days), and “RD 41” (105 days) rice varieties were planted twice a year. The pregerminated rice seeds were sown using the broadcasting method, and chemical fertilizers were applied using 46-0-0 (62.5 kg ha^−1^) and 16-20-0 (156.3 kg ha^−1^). Glyphosate (48% *w*/*v* SL) and alachlor (48% *w*/*v* EC) were applied to control weeds, whereas acephate (75% S) and chlorpyrifos (40% EC) were used for disease and insect control. A harvesting machine was used for rice harvesting. Rice straw and stubble were burnt in the field once a year after 20–25 days of sun-drying in August. After 1–3 days of burning, tillage was started for the second rice cultivation, which was harvested in November. As there was not sufficient water for a third rice cultivation, the field was left to fallow for approximately 5 months (December–April) to allow sufficient time for the natural decomposition of rice straw and stubble, and the first rice cultivation was started in May, with harvesting in September.

**Figure 1:**
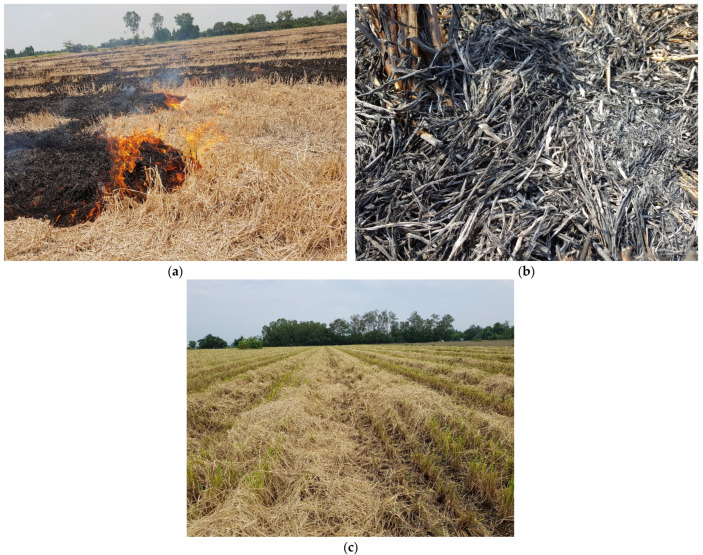
Rice straw and stubble burning practices: (**a**) during burning, (**b**) postburning, and (**c**) one year after burning. Photos were taken by Noppol Arunrat.

Five replicated plots were investigated in each field to assess the effects of rice straw and stubble burning on soil properties and bacterial communities, with an area of 5 × 5 m for each plot (Figure 1). Burning was conducted at 10.00–12.00 am and took approximately 98–130 s for each plot (the average burning speed was 6.5 s m^−2^). Air, fire, and soil temperatures as well as soil moisture were measured in each plot at three periods: before burning (19 August 2021), immediately after burning (19 August 2021), and 1 year after burning (27 August 2022). The fire temperature during burning was measured using an infrared thermometer. The soil temperature and soil moisture were measured at a depth of 5 cm with a Thermocouple Type K and a soil moisture meter, respectively. Each plot was burnt only once, meaning that there was no repeat burning even if rice straw and stubble remained, reflecting the current burning practice.

### 2.3. Sample Collection and Analysis

Soil samples before burning (preburning), immediately after burning (postburning), and 1 year after burning were obtained from five adjacent fields. In each field, five replicated plots at depths of 0–5 cm were investigated. In each plot, the soil samples were collected from five positions (four positions at the four corners of the plot and one in the center). Roots, grasses, stones, and residues were removed manually from the samples, which were then mixed to obtain one composite sample for each field. Approximately 1 kg of soil was placed into a plastic bag for the analysis of the soil physical and chemical properties. Additionally, 100 g samples of soil were placed into zip-lock plastic bags, cooled, and transported to the laboratory for the analysis of bacterial communities. To determine the soil bulk density, a 5.0 × 5.5 cm soil core was taken from each layer, and bulk density was measured after drying at 105 °C for 24 h.

Soil texture was determined using a hydrometer. The soil pH was measured with a pH meter using 1:1 suspensions of solids in water [18]. Electrical conductivity (EC_e_) was measured in saturation paste extracts using an EC meter [19]. The available calcium (Ca), magnesium (Mg), and potassium (K) levels were measured with atomic absorption spectrometry (NH_4_OAc pH 7.0 extraction) [20]. The available phosphorus (P) concentration was determined using the molybdate blue method (Bray II extraction) [21]. The ammonium nitrogen (NH_4_-N) and nitrate-nitrogen (NO_3_-N) levels were measured with the KCl extraction method, and total nitrogen (TN) was measured with the micro-Kjeldahl method. The cation exchange capacity (CEC) was analyzed using the NH_4_OAc pH 7.0 method. The organic carbon (OC) contents were determined following the method described by Walkley and Black [22]. The SOC stock was estimated using the following equation:

(1) SOC stock=OC×BD×L×10,000,

where *SOC* is the soil organic carbon stock (Mg C ha^−1^), *OC* is the organic carbon (%), *BD* is the soil bulk density (Mg m^−3^), and *L* is the soil thickness (m).

The STN stock was calculated using the following equation:

(2) STN stock=TN×BD×L×10,000,

where *STN* is the amount of soil total nitrogen (Mg N ha^−1^), *TN* is the total nitrogen (%), *BD* is the soil bulk density (Mg m^−3^), and *L* is the soil thickness (m).

### 2.4. DNA Extraction, Bacterial 16s Amplification, and Sequencing

The DNA was extracted from approximately 0.25 g of soil using a DNeasy PowerSoil Pro DNA Kit (Qiagen, USA). The hypervariable V3–V4 region was amplified with the 16s rRNA gene using primers 341F (5′-CCTAYGG-GDBGCWSCAG) and 805R (5′-GGACTAC-NVGGGTHTCTAAT-3′) [23]. Subsequently, the PCR products were sequenced using the Paired-end Illumina Miseq platform (2 × 250 bp) at the Omics Sciences and Bioinformatics Center of Chulalongkorn University (Bangkok, Thailand). All sequencing data associated with this study can be found in the National Center for Biotechnology Information (NCBI) under the BioProject accession number PRJNA881635.

### 2.5. Bacterial Taxonomic and Functional Identification

The bioinformatic analysis of the bacterial 16s rRNA gene was conducted on QIIME2 v. 2022.2 [24]. Raw sequence data were quality-filtered and merged, and chimera were removed using the DADA2-plugin [25]. Amplicon sequence variants (ASVs) with less than two sequence reads (singletons) were eliminated. Bacterial taxonomy was assigned using the Silva v. 138 database [26,27], and ASVs that were assigned to mitochondria or chloroplasts were removed. The remaining ASVs were then resampled and normalized to a minimum number of sequences from each sample using the rarefy plugin. This rarefied dataset was functionally assigned using PICRUSt2 [28] to predict the bacterial functions based on marker genes. The gene families for the bacterial sequences were annotated corresponding to the enzyme classification numbers (E.C. numbers). In this study, we highlighted 15 soil enzymes that potentially indicated soil health [29]. The E.C. numbers and names of these enzymes are presented in Supplementary Materials, Table S1.

### 2.6. Statistical Analysis

The soil properties before burning, immediately after burning, and after harvesting (1 year after burning) were compared with a one-way ANOVA and post hoc Tukey’s HSD tests. The visual graphics were generated with the ‘ggplot2’ package in the R environment (v. 4.0.2) [30]. The alpha diversity indices, which included the observed richness, Chao-1, Simpson, and Shannon indices, were computed and statistically compared among the sites using the ANOVA (for normal distribution data) or Kruskal–Wallis tests (for non-normal distribution data). Bacterial community and functional compositions were analyzed and visualized through a principal coordinate analysis (PCoA) based on the Bray–Curtis distance. The differences in compositions were tested using permutational multivariate analyses of variance (PERMANOVAs). A redundancy analysis (RDA) was employed to determine the influence of soil properties on soil bacterial community compositions, and the significance of the correlation between them was confirmed using the Mantel test.

## 3. Results

### 3.1. Soil Physical and Chemical Properties

No significant differences in soil moisture and soil temperature were found among the three periods (preburning, postburning, and 1 year after burning) (Table 1). At a depth of 5 cm, the soil moisture ranged from 45.1% to 48.4% in the preburning sites, and a slight decrease to 44.5–46.0% was detected in the postburning samples. A rise in soil temperature was measured at 25.9–26.8 °C after burning compared with preburning (25.7–26.5 °C). During burning, the fire temperature in the litter layer ranged from 415.5 to 469.5 °C.

There were no significant differences of soil physicochemical properties were detected between preburning and 1 year after burning (Table 2). At a depth of 0–5 cm in paddy soils, the bulk density, organic matter (OM), CEC, and soil texture showed no significant changes after burning. The soils had a significantly lower acidity as well as lower NO_3_-N levels after burning. Conversely, the burned soils showed higher levels of TN, NH_4_-N, available P, K, Ca, and Mg, and had higher ECe values (Table 2). At 1 year after burning, the soil pH, ECe, CEC, NH_4_-N, NO_3_-N, and available P, K, Ca, and Mg were higher than the initial values (preburning), whereas OM and TN were reduced and lower than the preburning values, but the significant differences were not found.

**Figure 2:**
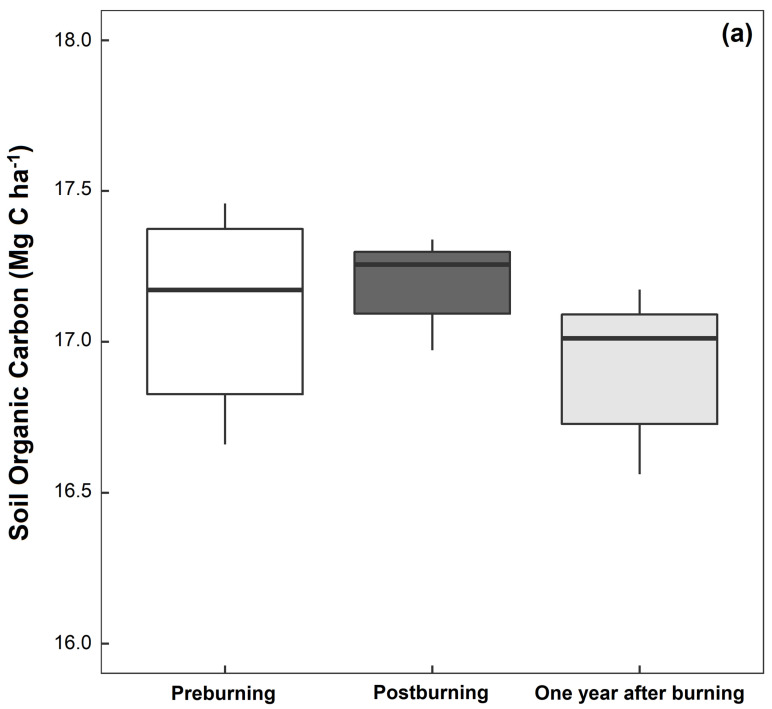

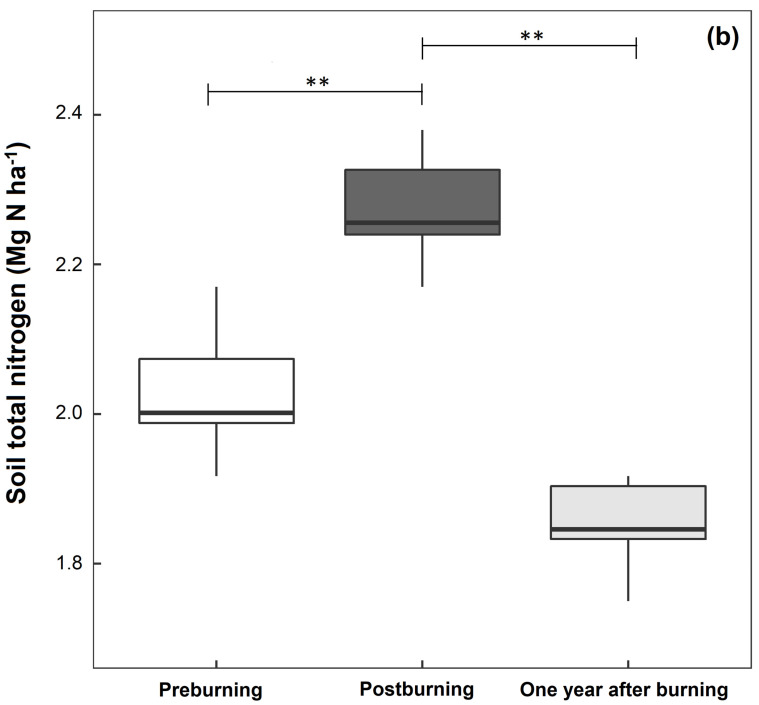
Soil organic carbon (SOC; (**a**) and soil total nitrogen (STN; (**b**) levels in paddy soils. ** denotes significant statistical differences (*p* ≤ 0.05).

The SOC stock was 17.10 ± 0.3 Mg C ha^−1^ in the preburning samples, which increased insignificantly to 17.19 ± 0.1 Mg C ha^−1^ after burning. A slightly declined SOC stock was observed at 1 year after burning, with 16.91 ± 0.23 Mg C ha^−1^, which was slightly lower than the preburning value (Figure 2a). A significantly higher STN stock, with an increase from 2.03 ± 0.09 Mg N ha^−1^ to 2.27 ± 0.081 Mg N ha^−1^, was also identified in the postburning soils. A significant reduction in the STN stock was detected at 1 year after burning (1.85 ± 0.07 Mg N ha^−1^) (Figure 2b).

### 3.2. Overview of the Sequencing Analysis

**Figure 3:**
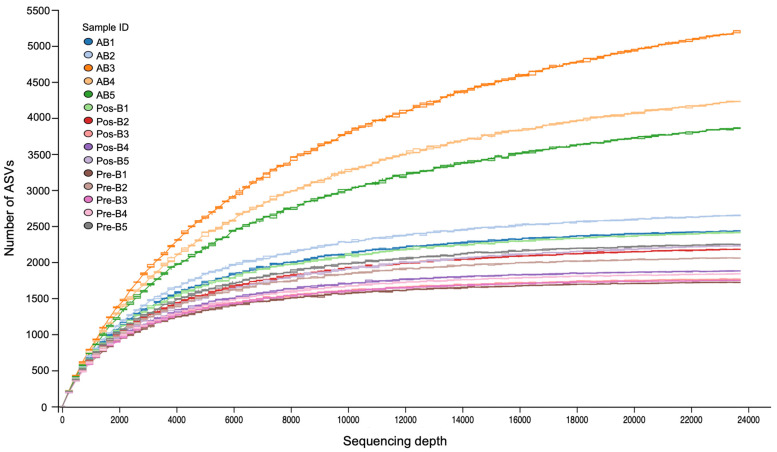
Rarefaction curves of all samples in rice fields at preburning (pre-B), postburning (pos-B), and 1 year after burning (AB).

A total of 616,815 (41,121 sequences/sample) clean sequences were obtained in this study. As shown in Figure 3, the rarefaction curves of all samples gradually flattened, indicating that the number of sequences obtained in this study could reflect the bacterial community in the study sites. Here, the sequences were grouped into 18,715 ASVs, which were then classified into 52 phyla, 119 classes, 245 orders, 312 families, and 543 genera.

### 3.3. Taxonomic Distribution

**Figure 4:**
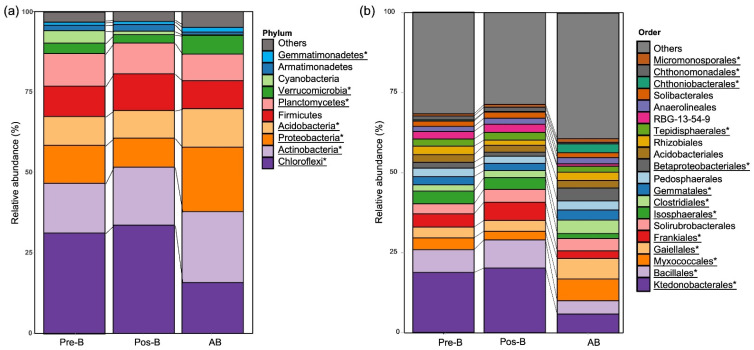
Stacked bar plot showing the relative abundances of the bacterial phyla (**a**) and orders (**b**) in rice fields at preburning (pre-B), postburning (pos-B), and 1 year after burning (AB). Asterisks beside the phylum name indicate statistical significance (*p* < 0.05).

As shown in Figure 4, whilst the most abundant taxa in the preburning and postburning samples were Chloroflexi (33%), followed by Actinobacteria (Pre-B = 15%; Pos-B = 18%), and Proteobacteria (Pre-B = 12%; Pos-B = 9%), those in the site 1 year after burning were Actinobacteria (22%), followed closely by Proteobacteria (20%) and Chloroflexi (16%) (Figure 4a). Overall, 11 phyla, 15 order, and 45 genera were indicated as differentially abundant taxa (*p* < 0.05; LDA score > 3). The ANOVA results showed that the relative abundances of 7 out of 10 abundant phyla (average relative abundance > 1%) were notably different among the study samples (Figure 4a). Chloroflexi abundance dramatically decreased by approximately 15–17% at 1 year after burning, whereas the abundance of Planctomycetes increased immediately after burning and then decreased, reaching a level similar to that before burning. The abundances of Actinobacteria, Proteobacteria, Verrucomicrobia, and Gemmatimonadetes significantly increased by 7%, 8%, 3%, and 1%, respectively, at 1 year after burning compared to the pre- and postburning soils. At the order level, significant changes were found to have occurred for several taxa (Figure 4b). The abundances of Ktedonobacterales, Bacillales, and Betaproteobacteria increased slightly immediately after burning, but significantly decreased by 13–14%, 3–5%, and 2–3%, respectively, at 1 year after burning. Myxococcales and Gaiellales abundances significantly increased by 3–4% at 1 year after burning, compared to the two other timepoints.

**Figure 5:**
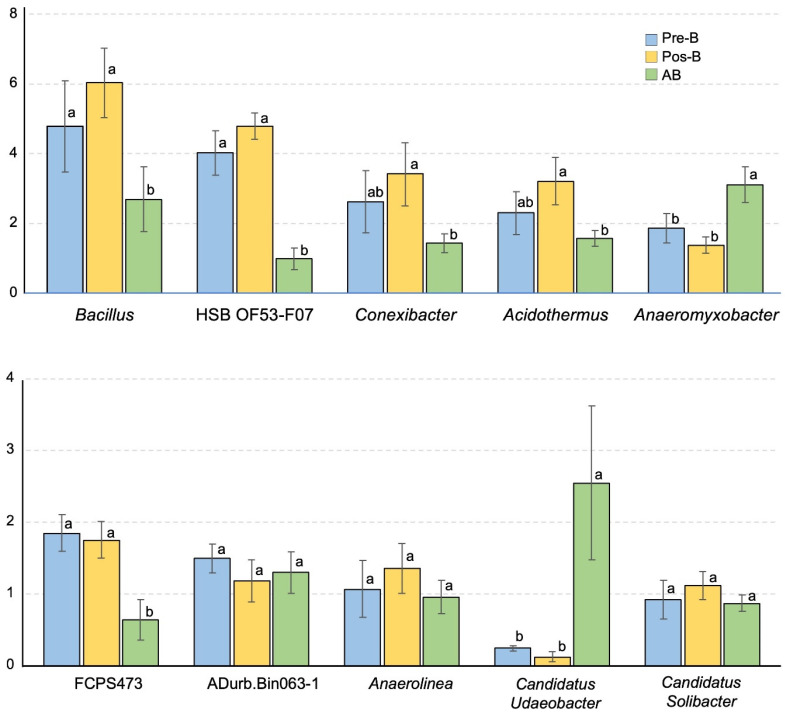
Bar plots for the most abundant genera in this study. Plots with different letters were statistically different. Pre-B—preburning; Pos-B—postburning; AB—1 year after burning.

Ten abundant genera (average relative abundance > 1%) were detected in this study. *Bacillus* and HSB OF53-F07 dominated at the pre- and postburning soils, accounting for 5% and 4% in the preburning sites and 6% and 4% in postburning soils. As shown in Figure 5, the abundances of these two taxa increased immediately after burning, but decreased significantly 1 year after burning. This trend was also found for *Conexibacter* and Acidothermus. On the other hand, the abundances of *Anaeromyxobacter* and *Candidatus* Udaeobacter increased significantly 1 year after burning, accounting for 3.1%, 2.7%, and 2.5%, respectively, of the taxa (Figure 5).

### 3.4. Bacterial Diversity, Community Compositions, and Correlations to Soil Properties

**Figure 6:**
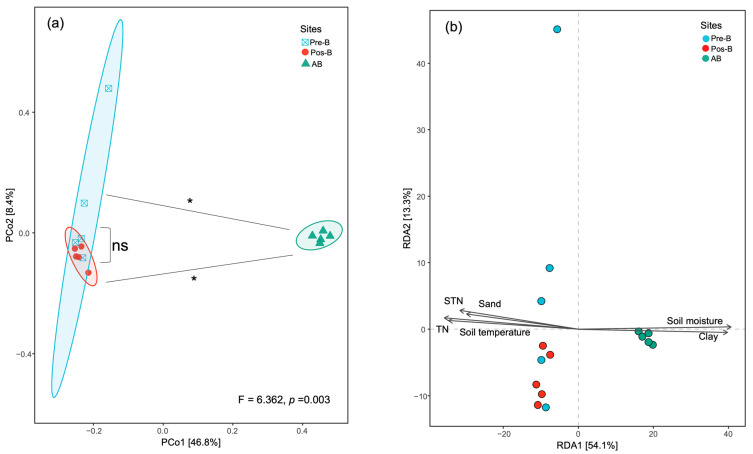
Bacterial community composition and correlations to soil properties. (**a**) Principal coordinate analysis (PCoA) ordination based on the Bray–Curtis distance, showing the community composition of bacteria detected in the study sites. (**b**) RDA ordination presents soil properties that significantly correlated with community composition. Significant parameters indicated with the Mantel test. Pre-B—preburning; Pos-B—postburning; AB—1 year after burning. * indicates statistically difference.

As shown in Table 3, all alpha diversity indices, including observed richness, Chao-1, Simpson, and Shannon indices, presented similar trends. The diversity indices did not change immediately after burning, but there were significant increases after 1 year. The beta diversity, presented by the PCoA ordination based on the Bray–Curtis distance, overlapped between the pre- and postburning samples, but these two groups were separate from those 1 year after burning (Figure 6a). According to the PERMANOVA results, the bacterial community compositions in the rice fields were significantly differed at 1 year after burning (Figure 6a).

As shown in Figure 6b, the redundancy analysis (RDA) revealed that the soil properties could explain 67.4% of the total variations in the bacterial community compositions. According to the Mantel test, the TN, STN, sand, clay, soil moisture, and soil temperature were significant soil parameters, shaping the bacterial communities (Table 4). Among these parameters, only clay and soil moisture presented a strong correlation (correlation coefficient > 0.7) (Table 4).

### 3.5. Predictive Functions

**Figure 7:**
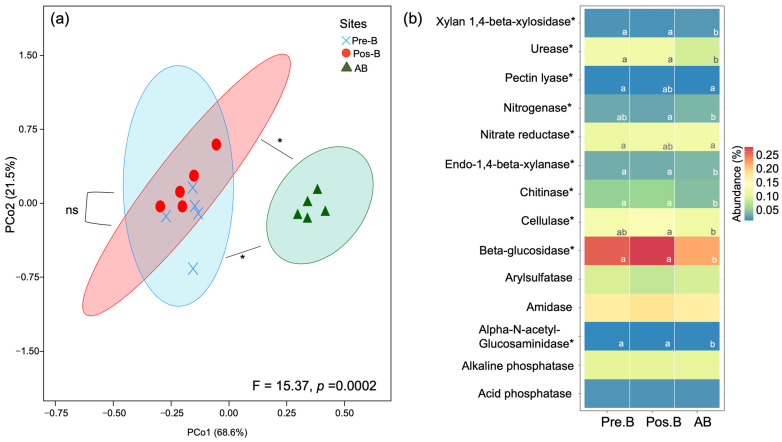
Bacterial functions predicted using PICRUSt2. (**a**) Principal coordinate analysis (PCoA) ordination, based on the Bray–Curtis distance, shows the functional composition of bacteria. (**b**) Heatmap shows the mean abundances of soil enzymes potentially produced by bacteria detected in the study sites. Pre-B—preburning; Pos-B—postburning; AB—1 year after burning. * indicates statistically difference.

We used PICRUSt2 to predict the functions of the bacterial community based on enzymatic genes. In total, 2372 predictive enzymes were detected across all samples. As shown in Figure 7a, PCoA, which explained 90.1% of the total functional composition, showed that the functional compositions of the bacterial communities found in preburning and postburning sites were largely similar, although they differed significantly 1 year after burning (PERMANOVA test, *p* > 0.05). In addition, 15 enzymes involved in the carbon, nitrogen, and phosphorus cycles were selected to highlight the potential enzyme activities in soils. The ANOVA results revealed that 10 out of 15 selected enzymes differed significantly among the study sites (Figure 7b). Whilst the abundances of β-glucosidase, chitinase, and urease were higher in pre- and postburning soils compared to the sites 1 year after burning, alpha-N-acetylglucosaminidase and endo-1,4-beta-xylanase presented inconsistent trends. Cellulase and nitrogenase levels increased 1 year after burning compared to the postburning soils. Pectin lyase and nitrate reductase showed similar levels at preburning and 1 year after burning.

**Figure 8:**
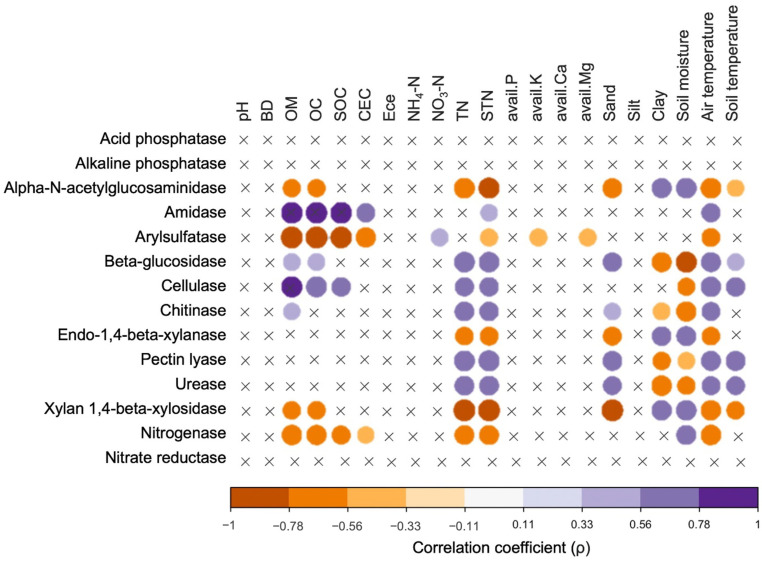
Spearman’s rank correlation between soil properties and soil enzymes predicted with PICRUSt2. X marks indicate insignificant correlation, whereas circles indicate a significant correlation (*p* < 0.05). Circle color corresponds to the correlation coefficient.

As shown in Figure 8, Spearman’s rank correlation analysis indicated correlations between soil properties and the selected soil enzymes. The parameters OM, OC, TN, STN, soil texture, moisture, and temperature were significantly correlated with several soil enzymes. Positive correlations were found, for example, for OM and amidase, cellulase, and chitinase, for SOC and amidase, cellulase, and TN, and for STN and β-glucosidase, chitinase, and urease. In contrast, negative correlations were found for these enzymes and the clay and soil moisture levels.

## 4. Discussion

### 4.1. Effects of Burning Rice Straw and Stubble on Soil Physicochemical Properties

Fire can alter the physical and chemical properties of soils through ash deposit. In our study, significant increases in pH, ECe, TN, NH_4_-N, and soil nutrients (available P, K, Ca, and Mg) were found (Table 2) after fire was used. An elevated soil pH following a fire was also reported in the meta-analysis of Ribeiro Filho et al. [31]. Granged et al. [32] also detected an increase in soil pH from 6.2 to 7.5 immediately after a fire. This increase could be explained by the loss of OH-groups from clay minerals, the formation of oxides, and the incorporation of ash into the soil, leading to an increase in the base cations in soils [33,34]. Kumar et al. [12] and Nigussie and Kissi [35] found increased ECe values after crop residue burning due to increased ash contents in the soil, along with higher available P levels after burning, possibly derived from the available P in ash. Kumar et al. [12] reported that the available P increased from 68.39 to 76.31 kg ha^−1^ after the use of fire. Nigussie and Kissi [35] detected an increase in the available P by 73.41% after burning.

Similarly, burning rice straw and stubble provided ash containing high K levels, resulting in an increase in the available K after burning, which was consistent with the results of the study by Gangwar et al. [36]. Burning facilitated the release of nitrogen from rice straw and stubble, increasing the TN content. Pellegrini et al. [37] and Parro et al. [38] also reported an increase in nitrogen after the use of fire. Ammonium (NH_4_-N) and nitrate (NO_3_-N) are the plant-available forms of nitrogen, and are generated via the decomposition of organic N compounds [39]. Previous studies [40,41,42] have reported high NH_4_-N concentrations after high-severity burning, whereas NO_3_-N did not form directly through heating [43], but was produced after burning through the nitrification of NH_4_-N [44]. This might explain the significant decrease in NO_3_-N of the paddy soils after burning (Table 2). According to Wan et al. (2001), NH_4_-N increases immediately after burning and returns to a preburning level after 1 year, whereas NO_3_-N can recover to preburning levels after 5 years. Covington et al. [45] also found that changes in NO_3_-N could not be detected immediately after a fire, but the levels increased 1 year after burning, exceeding the preburning levels.

Interestingly, the bulk density, OM, OC, CEC, and soil texture remained unaffected by the burning of rice straw and stubbles (Table 2), most likely because the fire was not severe enough to alter these soil properties. In contrast, Baldock and Smernik [46], Oguntunde et al. [47], and Ayodele et al. [48] detected a decreased bulk density due to the conversion of residues to a char form and the residue remaining from incomplete combustion. Although previous studies reported a decline in OM after burning [7,39,49], this was not the case in the present study. As shown in Figure 1, burning was practiced while the rice stubbles mostly stood in the fields, resulting in a lower impact of the fire on the soil surface. Due to the insignificant changes in OM and clay content after burning, the CEC was largely unaffected, as it is closely related to the OM and clay content. Similarly, Fonseca et al. [50] detected unchanged CEC levels after shrub burning in the northeast of Portugal. As shown in Figure 2, burnt paddy soils contained higher SOC stocks than unburnt paddy soils. The slight increase in the SOC stock after burning may have occurred as a result of the charred material and ash. However, a significant difference was not detected, indicating that burning rice straw and stubble did not alter the SOC stock. We, therefore, hypothesized that the fire was not severe enough to consume the soil carbon. This was in agreement with the findings of Neill et al. [51], who detected no significant changes in soil carbon levels in a prescribed burning in a Cape Cod oak–pine forest. In contrast, the STN stock increased significantly in the postburning soils (Figure 2), which was also observed in previous studies [52,53].

Due to remaining ash in the paddy fields, the soil pH, ECe, CEC, NH_4_-N, NO_3_-N, and available P, K, Ca, and Mg levels were higher 1 year after burning than before burning. On the other hand, the OM levels were lower after burning, most likely because of the slow decomposition of residues. A similar trend was observed for TN, which may have occurred due to plant uptake (Table 2). Burning rice straw and stubble resulted in temporary changes in SOC and STN stocks, as well as soil nutrient levels. Several studies have also reported that changes in soil nutrient levels were temporary and that, generally, the levels returned to those measured before burning [54,55,56].

### 4.2. Soil Bacterial Community Composition and Diversity Responses Immediately after Burning

Bacteria represent the most abundant and diversified population of microorganisms worldwide [57], and play an important role in the decomposition of OM and the cycling of nutrients in agricultural ecosystems [58,59]. Fire directly compromises the survival of soil microbial communities through soil heating [9]; in our study, straw burning caused a decline in soil microorganisms at the soil surface due to an increase in soil surface temperatures. Mickovski [10] reported that burning straw and stubble heated the soil temperature in the uppermost 0–3 cm to approximately 50–70 °C immediately after a fire. The heat resulted in the mortality of 77% and 9% of the heterotrophic microorganisms in the topsoil (0–5 cm) and the 5–10 cm layer, respectively. Biederbeck et al. [11] investigated the effects of burning cereal straw and reported that repeated burning in the field caused a reduction in the bacterial population by more than 50%. Kumar et al. [12] also reported that the soil temperature increased to 55 °C immediately after rice straw burning, resulting in a significant decrease in bacteria, fungi, actinomycetes, phosphate-solubilizing microorganisms, potassium-solubilizing microorganisms, and cellulose degraders after burning. The results of the abovementioned studies were, however, in contrast to our findings, namely, concerning the relative abundances of bacterial phyla, which did not significantly differ (*p* > 0.05) between preburning and postburning sites (Figure 4a). Our sites had a high initial soil moisture; therefore, the burning finished after only a short period of time (6.5 s m^−2^ on average), resulting in the fire not being severe enough to kill the soil microbial communities. Although the fire temperatures in the residue layer reached between 298.2 and 603.0 °C, the soil temperature after burning only increased by 1.6–3.8 °C compared to the preburning samples (Table 1). Moreover, it was hypothesized that whilst the fire could affect the soil microbial communities in the uppermost layer (0–1 cm), no effect was observed in the deeper layer (2–5 cm) because the high soil moisture contents may have had limited the heat transfer to the deeper soil layers. According to Busse et al. [60], heat transfer can decrease when the soil water content exceeds 20%. This was also supported by Whelan et al. [61], who reported that the prevalence of dormant soil microorganisms decreased with low soil moisture contents and high temperatures.

Previous studies reported that fire has a significant effect on the thin topsoil layer, such as the 0–1 cm layer [62], the 0–2.5 cm layer [11], and the 0–3 cm layer [63]. This could explain the insignificant difference between the soil microbial communities of the preburning and postburning sites observed here at a depth of 0–5 cm (Figure 4a). Similarly, Li et al. [64] investigated the responses of soil Acidobacteria to a wildfire disturbance in the topsoil (0–10 cm) and subsoil (10–20 cm), and found no significant differences in Acidobacterial α-diversity between these two soil layers across different fire severities. However, studies on the impacts of rice straw burning on soil microbial dynamics are still limited, since most studies have focused on wildfires or prescribed fires. Kumar et al. [12] investigated the responses of soil microbial communities to paddy straw burning in sandy loam soil. The authors discovered that soil microorganisms and microbial enzymes temporarily decreased after burning, before recovering 30–60 days after burning.

Chloroflexi were the dominant bacteria in both preburning and postburning fields, followed by Actinobacteria, Firmicutes, Planctomycetes, Proteobacteria, and Acidobacteria, although no significant difference was detected among the sites (Figure 4a). Previous studies also reported the dominance of these bacterial phyla in paddy soils [65,66,67,68]. Chloroflexi act as primary degraders of polysaccharides under the anaerobic conditions of rice fields, whereas Actinobacteria can also degrade OM under such conditions [69] and produce enzymes involved in carbon cycling for plant residue decomposition and carbon sequestration in soils [70]. Firmicutes comprise a range of thermophilic, antibiotic, and endospore-producing members, many of which are extremely resistant to desiccation, heat, and radiation, and can survive in extreme conditions [71]. Planctomycetes and Proteobacteria are important phyla involved in nitrification and denitrification processes in the soil [72,73]. Trivedi et al. [74] reported that Proteobacteria are classified as “copiotrophs” (R-strategists), which are more abundant in nutrient-rich conditions. Proteobacteria also play a key role in OM decomposition and produce several types of glycosyl hydrolases, thus, promoting plant growth [75]. Acidobacteria use diverse metabolic pathways in ecological processes, including biogeochemical cycles, biopolymer decomposition, and exopolysaccharide secretion [76]. Stinca et al. [77] investigated the soil in a beech forest 2 years after a wildfire, and found that the most abundant phyla (Proteobacteria, Acidobacteria, Bacteroidetes, Planctomycetes, Firmicutes, Gemmatimonadetes, and Chloroflexi) had remained relatively unaffected by the fire, except for Actinobacteria.

At the genus level, *Bacillus* and HSB OF53-F07 were the dominant taxa in both the preburning and postburning sites, but significant differences were not detected. Moreover, *Anaeromyxobacter* was more abundant in the preburning than postburning sites (*p* > 0.05), whereas *Acidothermus* was more abundant in the postburning sites than the preburning ones (*p* > 0.05) (Figure 5); *Acidothermus* is more resistant to higher temperatures than other bacteria. This was consistent with the findings of Mohagheghi et al. [78], who reported that the *Acidothermus* bacteria could remain active at high temperatures, with an optimum growth temperature of 55 °C. Furthermore, the *Acidothermus* genome contains genes that encode for thermostable enzymatic cellulose degradation.

Most soil microorganisms die at temperatures exceeding 50 °C due to changes in their cells and enzymes [79]. In the present study, β-glucosidase, chitinase, and urease were the dominate soil enzymes produced by the soil bacterial communities. However, the relative abundances of those enzymes did not differ significantly between preburning and postburning soils (Figure 7b). Stott et al. [80] reported that β-glucosidase is an important enzyme for the decomposition of crop residues and provides the energy source for heterotrophic bacteria.

### 4.3. Changes in Soil Physicochemical Properties and Soil Bacterial Community Composition and Diversity

Fires can promote the growth of soil bacteria with heat-resistance capacities and enhance those with potential fast-growth strategies [81]. Increased abundances of Actinobacteria, Proteobacteria, Verrucomicrobia, and Gemmatimonadetes were observed 1 year after burning, whereas the abundance of Chloroflexi decreased, and could not reach the initial value (Figure 4a). Although the Planctomycetes abundance increased immediately after burning, the relative abundance decreased and returned to that of the preburning soils. This was consistent with previous studies reporting that changes in soil physical and chemical properties after a fire could indirectly affect soil microbial communities [82,83,84]. Zhao et al. [17] reported that the relative abundances of Proteobacteria, Betaproteobacteria, and Actinobacteria significantly increased with increased levels of STN, available N, and available P. Our study also pointed out that STN, TN, sand, clay, soil moisture, and soil temperature significantly shaped the bacterial community composition (Table 2). The increased pH after burning (Table 2) was a crucial factor in determining microbial activities, as it led to changes in community composition and diversity.

At the genus level, the abundances of *Bacillus*, HSB OF53-F07, *Conexibacter*, and *Acidothermus* increased immediately after burning and then significantly decreased, whereas *Anaeromyxobacter* and Candidatus *Udaeobacter* dominated 1 year after burning (Figure 5). We hypothesized that *Bacillus*, HSB OF53-F07, *Conexibacter*, and *Acidothermus* were dominant genera in paddy soils, and may be highly resistant to heat, but with low growth rates. In contrast, *Anaeromyxobacter* and Candidatus *Udaeobacter* may have high potential growth rates and mainly occupy areas with increased soil nutrient levels after a fire. However, for a better understanding of the behavior of these bacteria, long-term studies are necessary.

*Bacillus* can promote plant growth through nitrogen fixation, the solubilization and mineralization of phosphorus, zinc, and iron [85]. *Conexibacter* can transform nitrate into nitrite via denitrification under limited oxygen conditions [86]. Seki et al. [87] reported that *Conexibacter* is a slow-growing microorganism and can survive in arid environments. *Acidothermus* is involved in cellulose degradation and enhances plant growth [88], whereas *Anaeromyxobacter* can fix and assimilate N_2_ to NH_4_N with the use of nitrogenase, and is involved in the reduction of N_2_O to N_2_ [89,90]. This could explain the increase in the abundance of *Anaeromyxobacter* (Figure 5) and in the levels of nitrogenase (Figure 7) 1 year after burning. Moreover, *Anaeromyxobacter* is an iron-reducing bacterium, using OM as the electron donors [91], and can denitrify NO_2_^−^ to NO through Fe^2+^ oxidation [92]. Candidatus *Udaeobacter* can secrete antibiotics in the soil and has the potential to remove trace gases, especially H_2_ [93]. Brewer et al. [94] revealed that Candidatus *Udaeobacter* is an aerobic heterotroph with numerous amino acid and vitamin transporters, minimizing the cellular architecture and sacrificing metabolic versatility to become dominant in the soil.

The cellulase and nitrogenase levels were increased 1 year after burning (Figure 7), most likely because the increased soil nutrient levels accelerated the microbial enzyme production. Cellulase is generally produced for cellulose decomposition mechanisms [95], whereas nitrogenase can reduce N_2_ to NH_3_ [96]. Soil enzymes play the most active part in all biochemical processes in soil. In the present study, positive correlations were found between OM and amidase, cellulase, and chitinase, and between SOC and amidase. Moreover, cellulase increased with increasing TN levels, and an increase in STN influenced β-glucosidase, chitinase, and urease (Figure 8). This led us to infer that soil enzymes were more related to OM levels and directly involved in OM mineralization, thus, affecting carbon and nitrogen cycles. To further reveal the effects of repeated fires on soil bacterial activities and soil properties, long-term studies are needed. Furthermore, it should be noted that the enzymatic results from this study based on the prediction tool, the actual measurements of the enzymatic activities, should be further investigated in the future.

## 5. Conclusions

Soil chemical properties (pH, ECe, NH_4_N, NO_3_N, TN, and available P, K, Ca, and Mg) of paddy soils significantly increased after burning. However, these values largely returned to prefire levels after 1 year. The most abundant taxa in preburning and postburning soils were Chloroflexi, Actinobacteria, and Proteobacteria. At 1 year after burning, Chloroflexi abundance decreased dramatically, whereas the abundances of Actinobacteria, Proteobacteria, Verrucomicrobia, and Gemmatimonadetes significantly increased. At the genus level, *Bacillus*, HSB OF53-F07, *Conexibacter*, and *Acidothermus* abundances increased immediately after burning, and then significantly declined, with lower levels 1 year after burning. *Anaeromyxobacter* and Candidatus *Udaeobacter* dominated at 1 year after burning. Fires tend to directly and indirectly affect soil microbial communities and functions. Direct effects are exerted through soil heating, killing some bacterial species, whereas indirect effects are due to changes in soil physicochemical properties. In this study, rice straw and standing stubble were burnt under high soil moisture conditions, and burning finished within a very short period of time, indicating that the fire was not severe enough to sufficiently heat the soil and kill the soil microorganisms.

## Figures and Tables

**Table 1:** Soil moisture and soil, air, and fire temperatures at the study sites (minimum–maximum).

Variables	Preburning	Postburning	One Year after Burning
Soil moisture (%)	45.1–48.4 a	44.5–46.0 a	49.0–55.3 a
Soil temperature (°C)	25.7–26.5 a	25.9–26.8 a	25.2–26.1 a
Fire temperature in the litter layer (°C)	415.5–469.5		

a letters denote significant statistical differences (*p* ≤ 0.05).

**Table 2:** Physicochemical properties of paddy soil samples before burning, after burning, and 1 year after burning.

Soil Properties	Preburning	Postburning	One Year after Burning
pH (1:1)	5.08 ± 0.03 a	6.89 ± 0.16 b	5.15 ± 0.01 a
Bulk density (g cm^−1^)	1.42 ± 0.01 a	1.40 ± 0.03 a	1.41 ± 0.01 a
Organic matter (%)	4.15 ± 0.05 a	4.22 ± 0.10 a	4.13 ± 0.03 a
Organic carbon (%)	2.39 ± 0.03 a	2.44 ± 0.07 a	2.38 ± 0.04 a
CEC (meq 100 g^−1^)	24.10 ± 0.68 a	27.16 ± 0.96 a	25.01 ± 0.51 a
ECe (dS m^−1^)	2.45 ± 0.05 a	5.24 ± 0.64 b	3.11 ± 0.04 a
NH_4_N (mg kg^−1^)	48.48 ± 1.59 a	58.06 ± 2.69 b	51.34 ± 1.88 a
NO_3_N (mg kg^−1^)	55.22 ± 2.69 a	19.90 ± 2.84 b	57.59 ± 3.66 a
Total nitrogen (%)	0.29 ± 0.01 a	0.32 ± 0.03 b	0.26 ± 0.03 a
Available P (mg kg^−1^)	50.52 ± 1.24 a	56.73 ± 3.28 b	53.36 ± 2.11 a
Available K (mg kg^−1^)	297.37 ± 19.35 a	505.91 ± 13.10 b	319.37 ± 21.33 a
Available Ca (mg kg^−1^)	1766.81 ± 96.58 a	2191.13 ± 65.86 b	1898.81± 77.11 a
Available Mg (mg kg^−1^)	288.67 ± 23.16 a	365.73 ± 21.06 b	312.67± 16.66 a
Sand (%)	36.20 ± 2.01 a	37.21 ± 1.98 a	35.68 ± 2.33 a
Silt (%)	36.19 ± 2.54 a	35.29 ± 2.66 a	34.91 ± 2.21 a
Clay (%)	27.61 ± 1.88 a	27.50 ± 2.69 a	29.41 ± 1.73 a
Texture	Clay Loam	Clay Loam	Clay Loam

a–b letters denote significant statistical differences (*p* ≤ 0.05).

**Table 3:** Alpha diversity indices.

Site	Observed Richness	Chao-1	Shannon	Simpson
Preburning	1936.80 ± 203.24 b	1954.39 ± 243.83 b	7.03 ± 0.10 b	0.9984 ± 0.0001 b
Postburning	2114.60 ± 273.81 b	2169.11 ± 315.25 b	7.09 ± 0.12 b	0.9984 ± 0.0002 b
One year after burning	3761.40 ± 1203.85 a	4176.56 ± 1547.81 a	7.66 ± 0.30 a	0.9991 ± 0.0002 a

a–b letters denote significant statistical differences (*p* ≤ 0.05).

**Table 4:** Correlations and significant values of the bacterial communities and soil properties determined with the Mantel test.

Soil Properties	Correlation Coefficient	*p*-Value
pH	0.0274	0.259
Bulk density	−0.1338	0.975
Organic matter	0.2153	0.056
Organic carbon	0.2357	0.052
Soil organic carbon	0.1802	0.071
CEC	−0.0739	0.712
Ece	−0.0194	0.452
NH_4_N	−0.1115	0.813
NO_3_N	0.0291	0.283
Total nitrogen	0.3780	0.010 *
Soil total nitrogen	0.4268	0.006 *
Available P	−0.1008	0.812
Available K	0.0292	0.260
Available Ca	−0.0704	0.758
Available Mg	−0.0252	0.510
Sand	0.3377	0.011 *
Silt	0.1602	0.094
Clay	0.7666	0.001 *
Soil moisture	0.7223	0.001 *
Air temperature	0.1176	0.142
Soil temperature	0.2259	0.028 *

* denotes significant statistical differences (*p* ≤ 0.05).

## Data Availability

Raw sequence data generated for this study are available in the sequence read archives (SRAs) of the National Center for Biotechnology Information (NCBI) under BioProject accession number PRJNA819169.

## Supplementary Materials

The following supporting information can be downloaded at: https://www.mdpi.com/article/10.3390/biology12040501/s1, Table S1: the enzyme classification numbers and their descriptions.
