# Supplementary material for: Effect of Rice Straw and Stubble Burning on Soil Physicochemical Properties and Bacterial Communities in Central Thailand
Source: Biology (Basel). 2023 Mar 26;12(4):501. doi: 10.3390/biology12040501 (PMC10135879; doi:10.3390/biology12040501)
Supplement: Supplementary file 1 [file biology-12-00501-s001.zip › biology-2280576-supplementary.pdf]

Supplementary material

Table S1. The enzyme classification numbers and their description.

E.C. number	Description
EC:3.1.3.2	Acid phosphatase
EC:3.2.1.1	Alpha-amylase
EC:3.2.1.50	Alpha-N-acetylglucosaminidase
EC:3.5.1.4	Amidase
EC:3.1.6.1	Arylsulfatase
EC:3.2.1.21	Beta-glucosidase
EC:3.2.1.4	Cellulase
EC:3.2.1.14	Chitinase
EC:3.2.1.8	Endo-1,4-beta-xylanase
EC:1.10.3.2	Laccase
EC:4.2.2.10	Pectin lyase
EC:1.11.1.7	Peroxidase
EC:3.5.1.5	Urease
EC:3.2.1.37	Xylan 1,4-beta-xylosidase
EC:3.1.3.1	Alkaline phosphatase
